# The association between trajectories of change in social functioning and psychological treatment outcome in university students: a growth mixture model analysis

**DOI:** 10.1017/S0033291723000363

**Published:** 2023-10

**Authors:** Phoebe Barnett, Rob Saunders, Joshua E. J. Buckman, Syed Ali Naqvi, Satwant Singh, Joshua Stott, Jon Wheatley, Stephen Pilling

**Affiliations:** 1 Centre for Outcomes Research and Effectiveness, Research Department of Clinical, Educational, & Health Psychology, University College London, London, UK; 2 National Collaborating Centre for Mental Health, Royal College of Psychiatrists, London, UK; 3 iCope – Camden & Islington Psychological Therapies Services, Camden and Islington NHS Foundation Trust, London, UK; 4 Barking & Dagenham and Havering IAPT services, North East London NHS Foundation Trust, London, UK; 5 Waltham Forest Talking Therapies – North East London Foundation Trust, London, UK; 6 Adapt Lab, Research Department of Clinical, Educational, & Health Psychology, University College London, London, UK; 7 Talk Changes: City & Hackney IAPT Service, Homerton University Hospital NHS Foundation Trust, London, UK; 8 Camden and Islington NHS Foundation Trust, London, UK

**Keywords:** Growth mixture modelling, mental health, social functioning, students

## Abstract

**Background:**

The transition to university and resultant social support network disruption can be detrimental to the mental health of university students. As the need for mental health support is becoming increasingly prevalent in students, identification of factors associated with poorer outcomes is a priority. Changes in social functioning have a bi-directional relationship with mental health, however it is not clear how such measures may be related to effectiveness of psychological treatments.

**Methods:**

Growth mixture models were estimated on a sample of 5221 students treated in routine mental health services to identify different trajectories of change in self-rated impairment in social leisure activities and close relationships during the course of treatment. Multinomial regression explored associations between trajectory classes and treatment outcomes.

**Results:**

Five trajectory classes were identified for social leisure activity impairment while three classes were identified for close relationship impairment. In both measures most students remained mildly impaired. Other trajectories included severe impairment with limited improvement, severe impairment with delayed improvement, and, in social leisure activities only, rapid improvement, and deterioration. Trajectories of improvement were associated with positive treatment outcomes while trajectories of worsening or stable severe impairment were associated with negative treatment outcomes.

**Conclusions:**

Changes in social functioning impairment are associated with psychological treatment outcomes in students, suggesting that these changes may be associated with treatment effectiveness as well as recovery experiences. Future research should seek to establish whether a causal link exists to understand whether integrating social support within psychological treatment may bring additional benefit for students.

## Introduction

Mental health conditions continue to be highly prevalent among university students: up to one third report an anxiety, mood or substance use disorder in their first year (Auerbach et al., 2016; Knapstad et al., 2021). The displacement and loss of social support when moving to university is a key risk factor for onset of these conditions (Bewick, Koutsopoulou, Miles, Slaa, & Barkham, 2010; Conley, Kirsch, Dickson, & Bryant, 2014; Conley, Shapiro, Huguenel, & Kirsch, 2020). Social support is crucial for adjustment to university life, and is associated with academic success and self-esteem (Conley et al., 2020) as well as depression (Alsubaie, Stain, Webster, & Wadman, 2019). Likewise, relationship difficulties, including with family and peers are the most commonly reported source of stress among students (Hurst, Baranik, & Daniel, 2013) and are associated with onset of depression and anxiety (Nola et al., 2021; Özdemir & Sağkal, 2021). Collectively, such social constructs can be considered as aspects of social functioning, which broadly describes an individual's ability to participate in society and their satisfaction with their social roles (Burns & Patrick, 2007).

In addition to its potential causative role, impaired social functioning can also be a consequence of poor mental health (Cacioppo, Hughes, Waite, Hawkley, & Thisted, 2006; Diehl, Jansen, Ishchanova, & Hilger-Kolb, 2018). This makes the nature and direction of associations between social functioning and mental health problems unclear. However, some research has attempted to understand the direction of effects, demonstrating that relationship conflict can precede psychological distress in young/emerging adults (Özdemir & Sağkal, 2021), and loneliness can predict subsequent changes in depressive symptoms in middle-aged adults, but not vice-versa (Cacioppo, Hawkley, & Thisted, 2010; Hawkley & Cacioppo, 2010). Therefore, social functioning and social support in general, and specifically loneliness and social isolation, could influence changes in mental health. This is further supported by evidence that social functioning is also associated with outcomes of mental health treatment (Buckman et al., 2021a; Wang, Mann, Lloyd-Evans, Ma, & Johnson, 2018). Given the emphasis on self-development and cultivating friendships while at university (Conley et al., 2014, 2020), this relationship could be stronger among students, who are considered to value informal contacts as sources of support when experiencing stress and mental health symptoms more so than non-students (D'Avanzo et al., 2012).

However, it is unclear whether students with lower social support would benefit from more intensive treatment or whether interventions to improve social aspects during the course of treatment are needed to mitigate their risk of poorer outcomes. Improvements in social functioning might act as a driver for subsequent improvement in mental health for students, as reductions in the distress that accompanies impairments in social functioning (Hurst et al., 2013) may enable students to focus on social roles and other developmental challenges of early adult life. Conversely, students who experience declines in social functioning during treatment may experience poorer treatment outcomes including deterioration of mental health (Hawkley & Cacioppo, 2010). Identifying these students with trajectories of decline in social function during treatment is likely to be of more use to clinicians over the use of baseline measures only, and may help clinicians to understand which patients are in need of additional intervention during the course of treatment more effectively (Saunders et al., 2019).

This study aimed to (1) identify different trajectories of change in social functioning in students treated for depression or anxiety in primary care mental health services, and (2) investigate associations between each of the identified trajectories of change and psychological treatment outcomes.

## Method

### Services

Participants were patients who attended one of eight Improving Access to Psychological Therapies (IAPT) services in the North Central and East London IAPT Service Improvement and Research Network (NCEL IAPT SIRN) (Saunders et al., 2020). This dataset has been used to explore a number of research questions regarding IAPT services (Saunders et al., 2020, 2021). For further details about IAPT services see online Supplementary Appendix 1.

### Sample

The initial sample consisted of 483 683 patients attending services between August 2008 and August 2020. Participants were then included if they:

1. Were aged 17–25
2. Reported being a student as their employment status at their initial assessment
3. Entered treatment and had data recorded at a minimum of three timepoints, as the analytic modelling technique (described below) required at least three observations to model trajectories of change
4. Scored above the cut-off for ‘caseness’ for depression or an anxiety disorder (See online Supplementary Appendix 2)
5. Had individual scores for relevant items on the Work and Social Adjustment Scale (WSAS; Mundt, Marks, Shear, and Greist (2002)).

Students were included in the analysis regardless of the type and intensity of treatment received. The final sample consisted of 5221 students. See online Supplementary Appendix 1 for a flow diagram.

### Measures

#### Social functioning

Items from the WSAS (Mundt et al., 2002) were used to examine changes in social functioning. Item 3 (a rating of how much a person's mental health problem impairs their social leisure activities, e.g. parties, pubs, outings, entertaining etc.) and item 5 (a rating of how much a person's mental health problem impairs their ability to form and maintain close relationships) were the focus of this analysis, as they are the most relevant items available for indicating impairment in the social aspects of university life (See online Supplementary Appendix 2 for more information and details of other items of the scale). Both items were self-rated on a scale of 0–8, with 0 representing no impairment and 8 representing severe impairment.

#### Additional measures

Remaining measures used in the analysis were measures of depressive symptoms, anxiety symptoms, phobic anxiety, the ‘problem descriptor’ [the mental health condition that is the agreed focus of treatment, matched to ICD-10 diagnoses; categorised as in previous studies using similar data (Buckman et al., 2021b; Saunders et al., 2019), see Table 1 for details], sociodemographic and other baseline variables (gender, age, ethnicity, sexual orientation, deciles of Indices of Multiple Deprivation, long-term health conditions, and psychotropic medication use), and treatment factors (number of high and low intensity treatment sessions received, waiting time between referral and assessment, waiting time between assessment and starting treatment, and the service attended). Full descriptions of measures, scales used and definitions are in Table 1.

**Table 1.** Measures, scales used, and definitions

Item	Questionnaire	Additional information/thresholds
Baseline mental health symptoms		
Depressive symptoms	Patient Health Questionnaire 9-item version [PHQ-9; (Kroenke, Spitzer, & Williams, 2001, 2003)]	Scores of at least 10 represent a case for depression. Changes of 6 or more represent reliable change.
Anxiety symptoms	The Generalised Anxiety Disorder Scale 7-item version (GAD-7; Spitzer, Kroenke, and Williams (2006))	Scores of at least 8 represent a case for generalised anxiety. Changes of 4 or more represent reliable change.
	‘Anxiety disorder specific measures’ (ADSMs)	ADSMs are used instead of the GAD-7 if the main identified problem is a specific anxiety disorder. The annual reports on the UK IAPT programme give more detail.
	*1. Agoraphobia: Mobility inventory* (Chambless, Caputo, Jasin, Gracely, and Williams, 1985)	Scores of at least 2.3 represent a case of agoraphobia. Changes of 0.7 or more represent reliable change
	*2. Health Anxiety: Health Anxiety inventory* (Salkovskis, Rimes, Warwick, and Clark, 2002)	Scores of at least 18 represent a case for health anxiety. Changes of 4 or more represent reliable change
	*3. Obsessive Compulsive Disorder* (*OCD*)*: Obsessive Compulsive inventory* (Foa, Kozak, Salkovskis, Coles, and Amir, 1998)	Scores of at least 40 represent a case for OCD. Changes of 32 or more represent reliable change.
	*4. Panic Disorder: Panic Disorder Severity Scale* (*PDSS;* Shear et al. (2001))	There is no threshold for a ‘case’ on the PDSS. Therefore GAD-7 scores are used to calculate IAPT outcomes for individuals with panic disorder as their main problem.
	*5. Post-Traumatic Stress Disorder* (*PTSD*)*: Impact of events Scale* (*IES-R;* Creamer, Bell, and Failla (2003))	Scores of at least 33 represent a case for PTSD. Changes of 9 or more represent reliable change
	*6. Social Anxiety Disorder: Social Phobia Inventory* (Connor et al., 2000)	Scores of at least 19 represent a case for social anxiety disorder. Changes of 10 or more represent reliable change
Phobic anxiety	IAPT Phobia scales (IAPT, 2011; NHS Digital, 2017)	These include three questions which assess avoidance of situations related to different phobias: agoraphobia, social phobia and specific phobia.
‘Problem descriptor’	Probable or confirmed diagnosis using ICD-10 codes	This is used to match participants based on their presenting symptoms to evidence-based treatment protocols. They are categorised following previous studies with similar data (Buckman et al., 2018; Saunders et al., 2021) as depression; mixed anxiety and depression; generalised anxiety disorder; OCD; PTSD; and phobic anxiety or panic.
Demographics and other baseline variables		
Demographics	–	Gender at point of referral, age, index of multiple deprivation (IMD) decile, sexual orientation and ethnicity (using UK census codes ‘White’, ‘Mixed’, ‘Asian’, ‘Black’, ‘Chinese’ and ‘other), all of which were self-reported.
Long-term health conditions	–	Dichotomous variable for participant report of presence of long-term health conditions (yes/no). The specific nature of conditions was not available.
Medication	–	Psychotropic medication use (prescribed and NOT taking, prescribed and taking, or not prescribed).
Treatment factors		
Number of low intensity (LI) and high intensity (HI) sessions	–	The number of sessions of each type (HI: face-to-face one-to-one (or some group work) sessions with a suitably trained therapist; LI: treatments with less intensive therapist input such as guided self-help and computerised CBT) received during the course of treatment were recorded
Time to assessment	–	The number of weeks between referral to the service and first assessment^a^
Time to treatment	–	The number of weeks between the assessment and first treatment session^a^
Length of episode	–	the number of weeks between assessment and last treatment session^a^
Service	–	The mental health service the patient was seen at

a Converted to weeks from days and winsorized at the top 99% due to a small number of extreme values.

#### Treatment outcomes

Eventual treatment outcomes consisted of dichotomous measures of reliable recovery, reliable improvement, deterioration, and attrition. These were defined as follows and have also been described elsewhere (Clark et al., 2018; NHS Digital, 2019):

*(Primary outcome) Reliable recovery:* Moving from ‘caseness’ (exceeding the pre-defined cut-off score for either the PHQ-9 or GAD-7 (or other relevant anxiety disorder specific measures; see Table 1) to ‘non-caseness’ after treatment alongside meeting criteria for reliable improvement

*Reliable improvement:* Reporting a reduction in symptoms scores which exceeds the reliable change threshold for either the PHQ-9 or GAD-7 [or relevant anxiety disorder specific measures (see Table 1 for detail on measures and thresholds)]

*Deterioration:* Reporting an increase in symptom scores which exceeds the reliable change threshold for either the PHQ-9 or GAD-7 (or relevant anxiety disorder specific measures)

*Attrition:* ‘Dropping out’ of the episode of treatment prior to completion of the number of treatment sessions planned. This measure only included participants who were not referred on for additional treatment (384 participants excluded)

*Time points:* Measures of social leisure activities and close social relationships from the first nine sessions (an initial assessment and eight treatment sessions) were used in the analysis. In Growth Mixture Modelling (GMM), the number of time points should be close to the sample mean (Lutz et al., 2005). Here, the mean number of sessions (timepoints) was 8.26 (s.d. = 4.60).

### Data analysis

Latent growth curve (LGC) analyses (Bollen & Curran, 2006) were conducted a first step in GMM (Jung & Wickrama, 2008; Wickrama, Lee, O'Neal, & Lorenz, 2021). GMMs were then built to identify different trajectories of change in impairment in social functioning, using the best fitting LGC model for the social leisure activities and close relationships measures. GMMs allow between-class and within-class variability and identify sub-groups of participants demonstrating statistically distinct trajectories of change on a given measure (Jung & Wickrama, 2008; Muthén, 2001; Muthén & Muthén, 2000). GMMs are particularly useful where each identified sub-population or ‘class’ is expected to have a trajectory which is not identical in every participant, as is often the case in mental health research (Muthén & Muthén, 2000; Rubel et al., 2015).

GMM model fit indices were compared to identify the model with best fit. Based on recommendations by Nylund, Asparouhov, and Muthén (2007), the Bayesian information criterion [BIC; (Schwarz, 1978)] and Bootstrap Likelihood Ratio test [BLRT; (McLachlan & Peel, 2000)] were considered the main metrics for model identification, although the Akaike information criterion [(AIC; (Akaike, 1987)], Vuong-Lo Medell Rubin Likelihood Ratio test [VLMR-LRT; (Lo, Mendell, & Rubin, 2001)] and Entropy (Jedidi, Ramaswamy, & DeSarbo, 1993) were also considered.

Once an optimal class solution was identified for each of social leisure activities and close relationships, the class with the highest conditional probability of membership was assigned and extracted per-participant. online Supplementary Appendix 3 provides additional information on LGC and GMM model identification procedures.

#### Associations between trajectories of social functioning and treatment outcomes

Logistic regression models were used to explore the association between trajectories of social functioning (WSAS-3 and WSAS-5 scores) and each of the outcome variables (reliable recovery, reliable improvement, deterioration and attrition) using Stata version 16 (StataCorp, 2019). Models were adjusted for potential confounders available in the dataset (full details on models and adjustments are provided in online Supplementary Appendix 3).

Analyses were conducted using imputed datasets, with sensitivity analyses conducted on complete data only (See online Supplementary Appendix 3 for details).

## Results

The majority of the sample identified as female (73.9%) and the mean age was 20.64 (s.d. = 2.20). Half the samples were White (50.1%) and the most commonly reported problem descriptor was depression (39.7%). Average baseline ratings of impairment on the WSAS-3 ‘Social leisure activities’ and the WSAS-5 ‘close relationships’ items were 4.40 (s.d. = 2.25) and 4.16 (s.d. = 2.36), respectively. This corresponds to a rating of ‘definite’ impairment due to mental health symptoms. Additional sample information is displayed in Table 2. By session nine, 1888 students (36.2%) still had individual WSAS item measures recorded (i.e. remained in treatment).

**Table 2.** Sample baseline characteristics and treatment outcomes

Continuous variables	*n*	M	s.d.
PHQ9	5220	15.09	5.21
GAD7	5219	13.62	4.27
WSAS-2	4889	3.36	2.33
WSAS-3	4889	4.40	2.25
WSAS-4	4888	3.54	2.42
WSAS-5	4889	4.16	2.36
Agoraphobia Item	5190	2.97	2.60
Social Phobia Item	5190	3.57	2.42
Specific Phobia Item	5189	2.38	2.58
Number LI sessions	5221	2.95	2.78
Number HI sessions	5221	5.26	5.41
Number total sessions	5221	8.26	4.60
Weeks-referral to assessment	5217	3.34	3.11
Weeks- assessment to treatment	5078	8.42	7.93
Age	5221	20.64	2.20
Categorical variables		*N*	%
Gender	Male	1346	25.78
	Female	3856	73.86
	Missing	19	0.36
Ethnicity	White	2617	50.12
	Mixed	446	8.54
	Asian	948	18.16
	Black	628	12.03
	Chinese	118	2.26
	Other	218	4.18
	Missing	246	4.71
IMD Decile	1	435	8.33
	2	1362	26.09
	3	1065	20.40
	4	645	12.35
	5	571	10.94
	6	412	7.89
	7	288	5.52
	8	210	4.02
	9	106	2.03
	10	47	0.90
	Missing	80	1.53
Sexual orientation	Heterosexual	3607	69.09
	Gay/Lesbian	177	3.39
	Bi-sexual	302	5.78
	Missing	1135	21.74
Medication	Prescribed not taking	242	4.64
	Prescribed and taking	1277	24.46
	Not prescribed	3409	65.29
	Missing	293	5.61
Long term condition	No	3526	67.53
	Yes	783	15.00
	Missing	912	17.47
Problem descriptor	Depression	2072	39.69
	Mixed A.D	287	5.50
	GAD	843	16.15
	OCD	200	3.83
	PTSD	129	2.47
	Other Phobia & Panic	308	5.90
	Social Phobia	352	6.74
	Unspecified anxiety	231	4.42
	Missing	799	15.30
Clinical outcomes	Reliable recovery	2398	45.93
	Reliable improvement	3738	71.60
	Deterioration	339	6.49
	Attrition	1487	28.48

A total of 34 986 WSAS3 and 34 985 WSAS5 scores were recorded for sessions 1–9 (WSAS3:M = 3.80, s.d. = 2.27; WSAS5:M = 3.58, s.d. = 2.31).

### LGC analyses

The quadratic model was the best fit for the data for both measures. Fit statistics and figures are available in online Supplementary Appendices 4 and 5.

### Growth mixture models

**Fig. 1.**
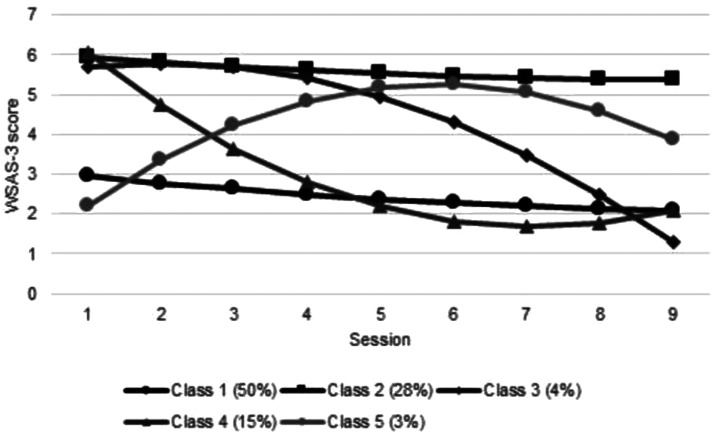
Social leisure activities trajectories.

**Fig. 2.**
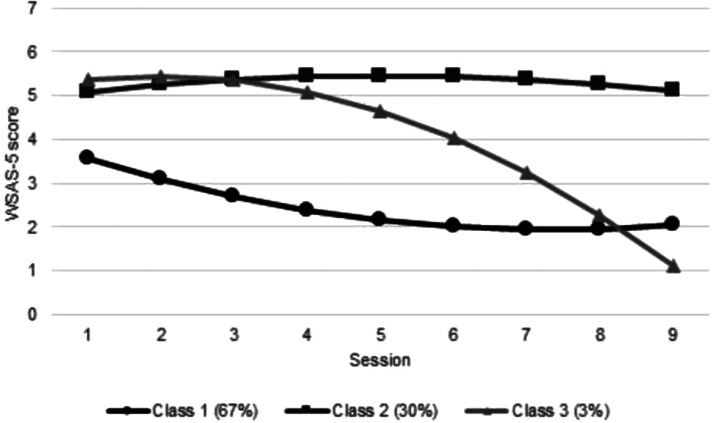
Close relationships trajectories.

GMM was performed using quadratic models separately on social leisure activities and close relationships measures across nine sessions. Model fit statistics and model selection results for both measures along with percentages of the sample assigned to each class are presented in online Supplementary Appendix 6. Trajectories of the 5-class solution for social leisure activities and the 3-class solution for close relationships are displayed in Figs 1 and 2.

#### Class trajectories of change in impairment in social functioning

For both measures (impairment in social leisure activities and impairment in close relationships), there were classes representing (1) mild (slight-definite impairment responses on average) impairment with some limited improvement over time (estimated mean change between session 1 and 9 was −0.86 for social leisure activities and −1.52 for close relationships) (2) severe (definite-marked impairment responses on average) impairment with limited change throughout (estimated mean change = −0.55 for social leisure activities and 0.01 for close relationships) and (3) severe impairment which remained stable until session 3 and then improved over time (estimated mean change = −4.43 for social leisure activities and −4.24 for close relationships). However, for social leisure activities, there were two additional classes: a fourth class of students who improved immediately following their first session but ended slightly more impaired than those in the delayed-improvement Class 3 (estimated mean change = −3.96), and a fifth who were the least impaired at session 1, became gradually more impaired and despite improving between sessions 6–9, were still ‘definitely’ impaired at that point (estimated mean change = 1.66).

Classes can be summarised as follows

#### WSAS-3: Social leisure activities

- Class 1:Mild impairment with limited change
- Class 2:Severe impairment with limited improvement
- Class 3:Severe impairment with delayed improvement
- Class 4:Severe impairment with early improvement
- Class 5:Mild impairment with deterioration

#### WSAS-5: Close social relationships

- Class 1:Mild impairment with limited change
- Class 2:Severe impairment with limited improvement
- Class 3:Severe impairment with delayed improvement

#### Comparison between social leisure activities and close relationships classes

There was considerable overlap between classes on the two items. Eighty-three per cent of those who showed mild impairment with limited change (Class 1) for social leisure activities were also in this class for close social relationships. Sixty-three per cent of those who showed severe impairment with limited improvement (Class 2) for social leisure activities were also in this class for close social relationships. However, there was only 30% overlap of those who showed delayed improvement in social leisure activities (Class 3) and those who showed the same trajectory in close relationships. Class 4 (social leisure activities), which also showed improvement but at an earlier stage also did not share many students with those in Class 3 for close relationships, but had 86% overlap with Class 1 for close social relationships. The largest proportion of students who showed deterioration in social leisure activities (Class 5) overlapped with Class 2 for close relationships (Table 3).

**Table 3.** Overlap in class assignment

Social leisure activities classes^a^	Close relationships classes^b^		
	1	2	3
1	2156	398	36
	83.24%	15.37%	1.39%
2	508	915	33
	34.89%	62.84%	2.27%
3	99	59	67
	44.00%	26.22%	29.78%
4	680	87	21
	86.29%	11.04%	2.66%
5	55	106	1
	33.95%	65.43%	0.62%

a Class 1: Mild impairment with limited change. Class 2: Severe impairment with limited improvement. Class 3: Severe impairment with delayed improvement. Class 4: Severe impairment with early improvement. Class 5: Mild impairment with deterioration.

b Class 1: Mild impairment with limited change. Class 2: Severe impairment with limited improvement. Class 3: Severe impairment with delayed improvement.

Note: percentages expressed as proportion of those assigned to WSAS-3 class.

### Class description

For both measures, those with mild impairment and limited change (Class 1) had the lowest scores on the majority of other baseline symptom measures. For social leisure activities, those with severe impairment and delayed improvement (Class 3) received the most high-intensity treatment sessions (M = 6.99, s.d. = 5.41) and those with severe impairment and early improvement (Class 4) received the least (M = 4.22, s.d. = 4.79). Deteriorators (Class 5) waited the longest between assessment to treatment (M = 10.29 weeks, s.d. = 9.35) while those in Class 1 waited the shortest (M = 7.99 weeks, s.d. = 7.64). For close relationships classes, those with severe impairment and limited improvement (Class 2) received the fewest low intensity (M = 2.77, s.d. = 2.84) and the greatest number of high intensity sessions (M = 6.36, s.d. = 5.84). Delayed improvers (Class 3) waited the longest between assessment and treatment (M = 9.34 weeks, s.d. = 8.75) while those in Class 1 waited the shortest (M = 8.16 weeks, s.d. = 7.81).

### Associations between social leisure activities class membership and treatment outcomes

The proportion of students experiencing reliable recovery and reliable improvement varied between classes: they were highest in Class 3 (64 and 88% respectively) and 4 (63 and 85% respectively), and lowest in Class 2 (25 and 60% respectively) and Class 5 (43 and 52% respectively). The latter two classes were also most likely to experience attrition (37 and 39% respectively) whereas those in Class 3 were least likely to experience attrition (12%). Class 5 were also most likely to deteriorate in treatment (19%) while Classes 3 and 4 were the least likely (approximately 3% in each) (online Supplementary Appendix 7a).

After adjusting for all covariates (service level variables, baseline severity and demographic factors), those in Class 2 for social leisure activities were less likely to reliably recover [OR 0.31, (95%CI 0.26–0.36)] and reliably improve [OR 0.37, (95%CI 0.31–0.44)] compared to those in Class 1, and were also significantly more likely to deteriorate [OR 3.22, (95%CI 2.41–4.29)] and drop out [OR 1.96, (95%CI 1.61–2.39)]. Classes 3 and 4 respectively were more likely to reliably recover compared to Class 1 [Class 3:OR 1.73, (95%CI 1.28–2.34); Class 4: OR 2.07, (95%CI 1.72–2.48)]. They were also more likely to reliably improve [Class 3:OR 1.87, (95%CI 1.22–2.88); Class 4:OR 1.80, (95%CI 1.43–2.26)]. Attrition was also less likely in Class 3 [OR 0.44, (95%CI 0.28–0.70)] and Class 4 [OR 0.64, (95%CI 0.51–0.80)] relative to Class 1. Class 5 were less likely to reliably recover [OR 0.28, (95%CI 0.19–0.42)] or reliably improve [OR 0.28, (95%CI 0.20–0.39)], more likely to deteriorate [OR 5.95, (95%CI 3.73–9.50)] and attrition was more likely relative to Class 1 too [OR 2.30, (95%CI 1.48–3.58)]. Class 5 had the highest odds of poor outcomes and the lowest odds of good treatment outcomes. Class 4 had the highest odds of reliable recovery while Class 3 had the highest odds of reliable improvement and the lowest odds of attrition (Table 4).

**Table 4.** Logistic regression analyses controlling for all variables of associations between class membership and treatment outcomes

WSAS item	Class (v. class 1)	Reliable recovery	Reliable improvement	Deterioration	Attrition
WSAS-3: Social leisure activities^ab^	Class 2	0.31 (0.26–0.36)	0.37 (0.31–0.44)	3.22 (2.41–4.29)	1.96 (1.61–2.39)
	Class 3	1.73 (1.28–2.34)	1.87 (1.22–2.88)	0.77 (0.33–1.80)	0.44 (0.28–0.70)
	Class 4	2.07 (1.72–2.48)	1.80 (1.43–2.26)	0.77 (0.48–1.21)	0.64 (0.51–0.80)
	Class 5	0.28 (0.19–0.42)	0.28 (0.20–0.39)	5.95 (3.73–9.50)	2.30 (1.48–3.58)
WSAS-5: Close relationships^a,c^	Class 2	0.28 (0.24–0.32)	0.32 (0.27–0.36)	3.69 (2.87–4.76)	2.34 (1.97–2.79)
	Class 3	1.72 (1.21–2.43)	1.68 (1.01–2.79)	0.41 (0.10–1.68)	0.52 (0.31–0.87)

* *N* = 5221 for reliable recovery, reliable improvement and deterioration. *N* = 4843 for attrition.

^a^Adjusted for number low intensity sessions, number high intensity sessions, weeks from referral to assessment, weeks from assessment to treatment, trust, PHQ9, GAD7, phobias, IMD, age, gender ethnicity, diagnosis, long term conditions, medication use, sexual orientation.

^b^Class 1: Mild impairment with limited change. Class 2: Severe impairment with limited improvement. Class 3: Severe impairment with delayed improvement. Class 4: Severe impairment with early improvement. Class 5: Mild impairment with deterioration.

^c^Class 1: Mild impairment with limited change. Class 2: Severe impairment with limited improvement. Class 3: Severe impairment with delayed improvement.

### Associations between close relationships class membership and treatment outcomes

Class 3 were most likely to experience reliable recovery (64%) and reliable improvement (89%) and Class 2 were least likely (26 and 59% for reliable recovery and reliable improvement, respectively). Similarly, Class 3 were least likely to experience deterioration (1.27%) and attrition (14%), and Class 2 were most likely (11 and 39% for deterioration and attrition, respectively) (online Supplementary Appendix 7b).

After adjusting for all covariates, relative to Class 1, Class 2 were less likely to reliably recover [OR 0.28, (95%CI 0.25–0.33)] or reliably improve [OR 0.32, (95%CI 0.27–0.36)] and both deterioration [OR 3.69, (95%CI 2.87–4.76)] and attrition [OR 2.34, (95%CI 1.97–2.79)] were more likely. Class 3 were more likely to reliably recover [OR 1.72, (95%CI 1.21–2.43)] or reliably improve [OR 1.68, (95%CI 1.01–2.79)], Attrition was also less likely in this group [OR 0.52, (95%CI 0.31–0.87)], but there was no evidence of a difference in deterioration compared to Class 1 (OR 0.41, (95%CI 0.10–1.68)].

Full results of all four regression models are shown in online Supplementary Appendix 8. Similar results were found in sensitivity analyses conducted on complete cases only (online Supplementary Appendix 9).

Logistic regression models comparing associations with treatment outcomes between classes who had similar baseline levels of social impairment (Classes 3 & 4 compared to Class 2) also showed that classes showing improvement in both measures were more likely to experience positive, and less likely to experience negative treatment outcomes (online Supplementary Appendices 10 and 11).

## Discussion

This study identified five different trajectories of change in impairment in social leisure activities, and three in impairment in close relationships in a sample of university students treated in routine mental health services. While over half of students experienced mild impairment in these measures, with impairment remaining relatively stable throughout treatment, about a quarter were severely impaired and remained this way throughout treatment. Just less than a quarter of students had impairments in social functioning that either improved or deteriorated over the course of treatment. Associations between trajectories of change and treatment outcome were also demonstrated. Firstly, in classes showing limited change in impairment in measures of social functioning, those who stayed severely impaired on the measure did not benefit from psychological therapy as much as those who remained mildly impaired, with odds of reliable recovery and improvement around a third of students who remained only mildly impaired. This supports previous research (Buckman et al., 2021a; Wang et al., 2018) and suggests being able to confide in close friends and spending social time with others throughout treatment could be an important facilitator of recovery. Second, improvement in social functioning from severe to mild impairment was associated with better treatment outcomes than maintaining mild impairment from start of treatment, additionally suggesting that positive changes in social functioning are associated with changes in depressive or anxiety symptoms through treatment. Importantly, classes showing such improvement also had around half the odds of attrition during treatment. This could suggest that perceptions of improvement in social aspects act as motivation to continue therapy more so than clinical symptom improvements. Third, although only observed in impairment in social leisure activities, deterioration in social functioning was associated with over five times the odds of deterioration in mental health symptoms, further supporting the fact that social functioning is a key aspect to consider in recovery and may be intrinsically linked with experiences of symptoms of mental health conditions.

While the current analysis found groups of students showing delayed (typically after session three) decrease in social functioning impairment which predicted positive outcomes, there was also a group of students showing rapid decreases in impairment in social leisure activities. This decrease began from session one, typically an assessment session, when it is unlikely that substantial intervention takes place. It is therefore possible that for some, seeking help in the first place is a major change point at which students begin participating in more social activities. This may in turn bolster future treatment gains. Furthermore, as students experiencing positive change in social functioning had higher odds of positive treatment outcome compared to students with mild but unchanging impairment, students may place particular importance on their ability to participate in the ‘social’ aspects of university and use this as a personal marker of recovery, which in turn could facilitate motivation and progress in therapy. Hawkins, Lambert, Vermeersch, Slade, and Tuttle (2006), for example, reported that participants who received feedback on progress in therapy had better outcomes compared to those who did not. Although patients in IAPT services do receive session-by-session feedback on progress, forming close friendships or socialising more where you previously could not may act as a more motivational form of explicit feedback, facilitating similar effects. This is supported by reports that young people favour more social compared to clinical markers of recovery, such as regaining their ‘place in world’ and ‘sense of self’ (Simonds, Pons, Stone, Warren, & John, 2014) and young adults consider reconnection with friends and family a vital part of recovery (Rayner, Thielking, & Lough, 2018).

However, a comparatively small subset of students experienced a large amount of change (positive or negative) in relation to their level of impairment in social functioning across the nine sessions of treatment. While 15% of students experienced rapid improvement in social leisure activity participation, only 4 and 3% of students showed slightly more delayed improvements in social leisure activities and close relationships, respectively, which could be associated with a response resulting from the therapy received. This could suggest that general psychotherapy provision could benefit from placing greater focus on supporting students to take more active roles in social aspects whilst at university. While the data provided here cannot imply causation, it does raise the question of whether more students would experience positive outcomes if the aspects of social functioning they consider most important were more central in individualisation of treatment plans.

Although the current study cannot be taken to prove that social functioning improvements drive improvements in symptoms (rather than vice-versa), development of interventions targeting social functioning (Haslam et al., 2019; Haslam, Cruwys, Haslam, Dingle, & Chang, 2016), have tested this hypothesis and shown significant improvements in depressive symptoms as a result in both young adults and adults. Furthermore, a systematic review of social interventions found that a range of strategies which encourage interactions with others may be effective in reducing depression in adults (Nagy & Moore, 2017).

The difference in the number of classes of trajectories between close relationships and social leisure activities is also of interest. For example, the majority of students who improved rapidly in social leisure activities were only mildly impaired in close social relationships at assessment. This could imply that having a support network of close friendships can encourage students to participate in social activities, even where impairment in other aspects of social functioning is substantial at the beginning of treatment. This is supported also by the fact that most students who deteriorated in social leisure activities were in the class that was severely impaired and remained so in close social relationships. Social networks may enhance the sense of control over desired outcomes in specific situations (such as during leisure activities) and encourage reinterpretation of events in a more positive light (Heaney & Israel, 2008; Thoits, 1995), possibly making participation seem less daunting in the face of symptoms of depression or anxiety. It follows that within the constraints of time and funding for student mental health support, integration of support with forming social group memberships to build social support networks may yield more benefit than more traditional social participation interventions which place focus on social activities rather than close social relationships (Haslam et al., 2016, 2019).

### Limitations

Despite the strengths of this analysis in shedding light on the association between social functioning and treatment outcome, the following limitations are noted. Two classes in this model were made up of only three per cent of the sample. Some have argued that small classes indicate that a solution with fewer classes is preferable (Spinhoven et al., 2016). However in larger samples, classes representing even smaller sample proportions can indicate a meaningful class of people (Mara & Carle, 2021). Three per cent corresponded to a noteworthy group size (156 students), and therefore it was determined that these classes remained clinically important.

In addition, the association between trajectories of social functioning and treatment outcome may instead be the result of classes of change being demonstrative of baseline severity across a range of symptomatic measures, which in turn predict treatment outcomes. To account for this, logistic regression analyses controlled for baseline depression and anxiety scores, and furthermore these scores did not definitively predict class trajectory (for example, baseline depression and anxiety scores were similar in classes that remained severely impaired and classes that improved). Online Supplementary analyses comparing classes with similar intercepts were also conducted to account for this, finding even stronger associations with outcomes in classes that improved social functioning compared to classes that remained impaired. Despite this, a major caveat of the current research is that the causal mechanism or direction between mental health symptoms and social functioning cannot be established. Furthermore, the uncertainty in class membership (measurement error) was not accounted for in the current approach, and future analyses should consider the use of approaches accounting for measurement error in class membership when looking at associations with treatment outcome.

Two further limitations are the possibility of bias from the selection of the sample and additional confounding variables. As NHS IAPT services are not the only available student mental health resource, we cannot argue that the current dataset is entirely representative of the student population- for example students with more or less severe symptoms may be more or less likely to seek help external to university-based services. Residual confounding from variables not available in the dataset could also have influenced associations between trajectories and outcomes, for example treatment expectancy (Delgadillo, Moreea, & Lutz, 2016) and duration of mental health disorder (Lorenzo-Luaces, Rodriguez-Quintana, & Bailey, 2020) are both prognostic in adults and may also influence outcomes in students. As there was limited additional information on student backgrounds, for example year or level of study within the dataset, it is also possible that these variables impacted results.

Finally, this analysis used a crude measure of ‘social functioning’ (the WSAS), and used single items in place of the total score to target aspects of social functioning most likely to impact students. This may have limited variance within available scores, as well as the validity of the measure. Also, the WSAS measures the extent of impairment in social functioning experienced as a result of mental health symptoms. Although within the IAPT dataset the WSAS is the best available indicator of social functioning, results should be contextualised within the limitations of this measure in its connection to mental health symptoms, as results are more likely to be correlated with mental health outcomes than other measures of social functioning may have been. However, it remains noteworthy that even in a scale linked to mental health symptoms, improvements in social functioning from a higher level of impairment showed a stronger association with positive treatment outcomes than remaining relatively unimpaired, as levels of impairment in later sessions were similar.

### Implications for future research

This study leads to some important avenues for future work. Establishing improved social functioning measures, particularly relating to specific aspects such as loneliness, or motivation to participate in social activities would allow for a more nuanced understanding of how social changes throughout treatment are related to clinical improvement. Qualitative research exploring patient experiences of how changes in social functioning relate to treatment experiences (and vice-versa) could further understanding of how symptomology and social functioning interact.

Future quantitative work should seek to establish whether social changes act as a causal mechanism for making psychological treatment more effective. This could be through use of cross-lagged panel models, which help to untangle the timing of changes in measures to establish which of two factors occurs first. While research has shown that loneliness in the preceding year predicts symptoms of depression using this technique (Cacioppo et al., 2010), establishing whether social functioning changes occur prior to symptomatic improvement during treatment could further elucidate how adaptations to treatment could enhance recovery for students. Additional research using RCTs comparing interventions with and without support in developing social networks during university could also establish whether such efforts should be integrated into models of support.

## Conclusions

Overall, students experience different trajectories of change in impairment in social functioning during the course of mental health treatment, and these are differentially associated with treatment outcomes. There may therefore be a link between social functioning and how effective psychological treatment is for a given individual, as well as they personal recovery experience. Future work should look to establish whether the addition of support to improve social functioning within therapy can further contribute to positive outcomes of treatment for students.

## Supplementary material

For supplementary material accompanying this paper visit https://doi.org/10.1017/S0033291723000363. click here to view supplementary material
