# Supplementary material for: The association between trajectories of change in social functioning and psychological treatment outcome in university students: a growth mixture model analysis
Source: Psychol Med. 2023 Mar 6;53(14):6848–58. doi: 10.1017/S0033291723000363 (PMC10600814; doi:10.1017/S0033291723000363)
Supplement: Supplementary file 1 [file S0033291723000363sup001.docx]

Supplementary Appendices

# Appendix 1: Service information and Participant Inclusion

IAPT services are primary care and community-based mental health services within the English National Health Service (NHS) that deliver evidence-based psychological therapies for depression and anxiety disorders in a stepped-care model as recommended by national clinical guidelines (Clark, 2018). As sessional outcome measures are mandated within IAPT services, pre- and post- intervention data are available for more than 98% of episodes, along with session-by-session monitoring of a range of symptom and functioning measures (Clark et al., 2018).

## Flow diagram of participant inclusion

483,683 original referrals

99,621 participants remaining

Age outside 17-25 at first assessment

<17: 1,930 participants excluded

>25: 382,103 participants excluded

Age missing: 29 participants excluded

Employment status not marked as student (e.g. homemaker, retired, unemployed, employed): 84,730 participants excluded

14,891 participants remaining

Did not enter treatment (at least two treatment sessions): 7,232 participants excluded

Did not record at least 3 timepoints: 979 participants excluded

6,680 participants remaining

Not scoring above the cut off for “caseness” of anxiety or depression: 576 participants excluded

6,104 participants remaining

No individual WSAS item measures recorded (some patients only had total WSAS scores recorded in the database): 883 participants excluded

5,221 participants remaining

# Appendix 2: Additional information on the measurement of social functioning-The Work and Social Adjustment Scale

The WSAS is a self-report scale of functional impairment attributable to an identified problem (Marks, 1986; Mundt, Marks, Shear, & Greist, 2002), and has been used frequently to study the effects of anxiety and depression treatment. The WSAS total score constitutes a measure of overall functional impairment, including in employment, home management, social leisure activities, private leisure activities, and close relationships, however the “employment” item is not scored for people who are not working, making the total score a less useful measure in exploring social functioning in students. Items are self-rated on a scale of 0-8, with 0 representing no impairment and 8 representing severe impairment.

### Social leisure activities

The third item of the WSAS scale asks patients to rate how much their mental health problem impairs their social leisure activities (with other people, such as parties, bars, clubs, outings, visits, dating and home entertaining).

### Close relationships

The fifth item of the WSAS scale asks patients to rate how much their mental health problem impairs their ability to form and maintain close relationships with others, including those they live with.

# Appendix 3: Additional Analysis information

## Latent growth curve (LGC) analysis

LGC analyses (Bollen & Curran, 2006) were conducted first using MPlus (Muthén & Muthén, 1998-2017) separately for the social leisure and close relationships measures in order to establish the best fitting growth model, regarding the form of change, for the data. Linear and quadratic curves were fitted and compared using the root mean squared error of approximation (RMSEA; (Browne & Cudeck, 1992; Steiger & Lind, 1980)), the standardised root mean squared residual (SRMR; Hu and Bentler (1999)), the comparative fit index (CFI; (Bentler, 1990)) and the Tucker-Lewis Index (TLI; (Bentler & Bonett, 1980; Tucker & Lewis, 1973)). Values of <0.08 indicate a reasonable model fit on the RMSEA and SRMR while values of >0.95 and >0.97 represent good and excellent fit, respectively on the CFI and TLI.

## Growth mixture model fit indices

The Vuong-Lo Medell Rubin Likelihood Ratio test (VLMR-LRT; (Lo, Mendell, & Rubin, 2001)) and Bootstrap Likelihood Ratio test (BLRT; (McLachlan & Peel, 2000)) compare the model to a model with one fewer class (i.e. the k compared to the k-1 model) and give the probability that the k-class model significantly improves the model fit (Nylund, Asparouhov, & Muthén, 2007). Higher values of entropy indicate higher accuracy and therefore distinct separation between classes while lower AIC and BIC values indicate better fitting models.

## GMM procedure

In line with recommendations where no prior hypothesis exists (Wickrama, Lee, O'Neal, & Lorenz, 2021), each GMM was conducted first with two classes, and subsequently with the number of classes increased by 1 until additional classes did not improve model fit. Models which failed to converge as a result of negative residual variances or correlations greater than one were also disregarded, as this indicates poor model fit (Nylund et al., 2007). To prevent the identification of local solutions 800 random starts and 80 final iterations were used. In all analyses the best log likelihood was replicated, suggesting that the global solution was obtained.

## Associations between trajectories of social functioning and treatment outcomes: Model information

Model 1 explored the univariable association between class membership and each of the outcomes in turn, Model 2 additionally adjusted for treatment-related variables (number of low intensity sessions, number of high intensity sessions, weeks from referral to assessment, weeks from assessment to treatment, and service), Model 3 additionally adjusted for baseline symptom severity (PHQ-9, GAD-7, and the three IAPT phobia scale items) and Model 4 additionally adjusted for other socio-demographic and clinical factors (IMD decile, age, gender, sexual orientation, ethnicity, problem descriptor, presence of long-term health conditions, and medication prescription). See Appendix 2 for full details of potential confounders.

## Missing data

Missing WSAS-3 and WSAS-5 data were handled using Full Information Maximum-Likelihood through the Expectation Maximisation algorithm (Dempster, Laird, & Rubin, 1977) during LCG and GMM analyses in Mplus.

Missing data on continuous covariates in logistic regression models were imputed using multiple imputations with chained equations (MICE) in Stata. Missing data for categorical covariates were dummy coded as “missing” to ensure that participants with missing information on these variables were included in the analysis. Fifty imputed datasets were created. Regression analyses were conducted using these imputed datasets, with sensitivity analyses conducted on complete data only.

| Appendix 4: LGC model fit statistics and estimates of variance | | | | | | | | |
| --- | --- | --- | --- | --- | --- | --- | --- | --- |
| **Measure** | **Model** | **RMSEA**  **(90% CI)** | **CFI** | **TLI** | **SRMR** | **Intercept variance (p)** | **Slope variance (p)** | **Quadratic variance (p)** |
| WSAS-3: Social leisure activities | Linear | 0.062 (0.058-0.065) | 0.96 | 0.96 | 0.079 | 3.127 (<0.001) | 0.064 (p<0.001) |  |
|  | Quadratic | 0.034 (0.031-0.038) | 0.99 | 0.99 | 0.020 | 3.150 (p<0.001) | 0.382  (p<0.001) | 0.004 (p<0.001) |
| WSAS-5: Close relationships | Linear | 0.058 (0.054-0.061) | 0.97 | 0.97 | 0.072 | 3.317 (p<0.001) | 0.058 (p<0.001) |  |
|  | Quadratic | 0.034 (0.031-0.038) | 0.99 | 0.99 | 0.200 | 3.142 (p<0.001) | 0.318 (p<0.001) | 0.004 (p<0.001) |

# Appendix 5: LGC models

| Appendix 6: Model fit statistics and results | | | | | | | | |
| --- | --- | --- | --- | --- | --- | --- | --- | --- |
| **Measure** | **Class solution** | **Log-Likelihood** | **AIC** | **BIC** | **Entropy** | **VLMR-LRT p-value** | **BLRT p-value** | **% individuals per class** |
| WSAS 3: social leisure activities | 2 | -64834.99 | 129714 | 129858 | 0.555 | <0.001 | <0.001 | 75/25 |
|  | 3 | -64752.06 | 129556 | 129727 | 0.525 | <0.001 | <0.001 | 19/53/27 |
|  | 4 | -64695.29 | 129451 | 129647 | 0.561 | 0.009 | <0.001 | 5/29/12/54 |
|  | 5 | -64636.23 | 129340 | 129564 | 0.611 | 0.001 | <0.001 | 50/28/4/15/3 |
|  | 6* | -64607.05 | 129290 | 129539 | 0.621 | 0.043 | <0.001 | 49/1/5/27/3/14 |
| WSAS 5: Close relationships | 2 | -65068.61 | 130181 | 130326 | 0.578 | 0.135 | <0.001 | 70/30 |
|  | 3 | -65014.37 | 130081 | 130251 | 0.624 | 0.022 | <0.001 | 67/30/3 |
|  | 4* | -64933.49 | 129927 | 130124 | 0.63 | <0.001 | <0.001 | 30/3/47/20 |
| *Model failed to converge | | | | | | | | |

## Social Leisure Activities

GMMs for the social leisure activities measure were compared for 2, 3, 4, 5, and 6-class models. However, the 6-class model resulted in correlations greater than one between the intercept and slope, the intercept and quadratic, and the slope and quadratic, indicating poor model fit. The 6-class model was therefore disregarded and the 5-class model was selected as the most appropriate model given it demonstrated better fit than the solutions with less classes.

## Close Relationships

GMMs for the close relationships measure were compared for 2, 3, and 4-class models. As with WSAS3, issues with correlations greater than one between the intercept and the slope, the intercept and the quadratic and the slope and quadratic, indicating poor model fit. The 4-class model was therefore disregarded. Although there was a non-significant VLMR-LRT p-value for the 2-class model, the 3-class model was a better fit for the data, indicated by reduced BIC and significant BLRT p-values. The 3-class model was therefore selected as the most appropriate model for the data.

# Appendix 7: Class descriptions

## Appendix 7a) Descriptive statistics of classes of WSAS3 trajectories

| **Appendix 5.2a: Characteristics of WSAS-3 trajectory classes** | | | | | | | | | | | | | | | | | | |
| --- | --- | --- | --- | --- | --- | --- | --- | --- | --- | --- | --- | --- | --- | --- | --- | --- | --- | --- |
|  | | **WSAS-3 Social Leisure** | | | | | | | | | | | | | | | | |
|  |  | **Class 1 (n=2,590)** | | | **Class 2 (n=1,456)** | | | **Class 3 (n=224)** | | | **Class 4 (n=788)** | | | **Class 5 (n=162)** | | | **F (df=4)** | **p** |
| **Continuous measures** | | **n** | **M** | **SD** | **n** | **M** | **SD** | **n** | **M** | **SD** | **n** | **M** | **SD** | **n** | **M** | **SD** |  |  |
| PHQ-9 | | 2,590 | 13.53 | 4.93 | 1,456 | 17.00 | 4.89 | 224 | 16.47 | 5.35 | 788 | 16.21 | 5.03 | 162 | 15.45 | 5.19 | 132.44 | <0.001 |
| GAD-7 | | 2,589 | 12.68 | 4.15 | 1,456 | 14.63 | 4.16 | 224 | 14.61 | 4.32 | 788 | 14.53 | 4.16 | 162 | 13.81 | 4.22 | 66.98 | <0.001 |
| WSAS-2 | | 2,544 | 2.74 | 2.10 | 1,436 | 4.15 | 2.35 | 222 | 3.82 | 2.40 | 784 | 3.88 | 2.33 | 162 | 3.07 | 2.48 | 106.66 | <0.001 |
| WSAS-3 | | 2,544 | 2.86 | 1.58 | 1,436 | 6.09 | 1.42 | 222 | 5.92 | 1.58 | 784 | 6.37 | 1.19 | 162 | 1.70 | 1.39 | 1703.55 | <0.001 |
| WSAS-4 | | 2,543 | 2.88 | 2.16 | 1,436 | 4.33 | 2.43 | 222 | 4.05 | 2.61 | 784 | 4.25 | 2.44 | 162 | 2.83 | 2.39 | 119.50 | <0.001 |
| WSAS-5 | | 2,544 | 3.36 | 2.17 | 1,436 | 5.14 | 2.14 | 222 | 4.81 | 2.35 | 784 | 4.80 | 2.33 | 162 | 3.65 | 2.39 | 178.48 | <0.001 |
| Agoraphobia item | | 2,571 | 2.32 | 2.34 | 1,447 | 3.85 | 2.64 | 224 | 3.44 | 2.82 | 786 | 3.37 | 2.69 | 162 | 2.76 | 2.55 | 96.13 | <0.001 |
| Social phobia item | | 2,571 | 2.64 | 2.06 | 1,447 | 4.79 | 2.32 | 224 | 4.19 | 2.43 | 786 | 4.19 | 2.41 | 162 | 3.43 | 2.54 | 241.23 | <0.001 |
| Specific phobia item | | 2,571 | 1.96 | 2.34 | 1,446 | 2.94 | 2.74 | 224 | 2.49 | 2.71 | 786 | 2.63 | 2.74 | 162 | 2.46 | 2.68 | 37.03 | <0.001 |
| Number LI sessions | | 2,590 | 2.99 | 2.72 | 1,456 | 2.80 | 2.88 | 225 | 2.93 | 3.03 | 788 | 3.12 | 2.67 | 162 | 3.05 | 3.02 | 1.95 | 0.100 |
| Number HI sessions | | 2,590 | 4.69 | 5.10 | 1,456 | 6.46 | 5.97 | 225 | 6.99 | 5.41 | 788 | 4.22 | 4.79 | 162 | 6.17 | 5.46 | 40.20 | <0.001 |
| Weeks-referral to assessment | | 2,587 | 3.40 | 3.14 | 1,456 | 3.32 | 3.12 | 225 | 3.34 | 3.27 | 787 | 3.16 | 2.93 | 162 | 3.20 | 3.19 | 1.03 | 0.389 |
| Weeks-assessment to treatment | | 2,526 | 7.99 | 7.64 | 1,414 | 8.79 | 7.98 | 221 | 9.50 | 8.72 | 760 | 8.46 | 8.11 | 157 | 10.29 | 9.35 | 5.87 | <0.001 |
| Age | | 2,590 | 20.71 | 2.23 | 1,456 | 20.55 | 2.18 | 225 | 20.51 | 2.19 | 788 | 20.62 | 2.18 | 162 | 20.54 | 2.18 | 1.57 | 0.178 |
| **Categorical Measures** | | **N** | **%** | | **N** | **%** | | **N** | **%** | | **N** | **%** | | **N** | **%** | | **X2 (df)** | **p** |
| Gender | Male | 687 | 26.53 | | 341 | 23.42 | | 58 | 25.78 | | 209 | 26.52 | | 51 | 31.48 | | 10.77 (8) | 0.215 |
|  | Female | 1,894 | 73.13 | | 1,107 | 76.03 | | 167 | 74.22 | | 577 | 73.22 | | 111 | 68.52 | |  |  |
|  | Missing | 9 | 0.35 | | 8 | 0.55 | | 0 | 0.00 | | 2 | 0.25 | | 0 | 0.00 | |  |  |
| Ethnicity | White | 1,382 | 53.36 | | 677 | 46.50 | | 115 | 51.11 | | 370 | 46.95 | | 73 | 45.06 | | 69.16 (24) | <0.001 |
|  | Mixed | 236 | 9.11 | | 114 | 7.83 | | 18 | 8.00 | | 67 | 8.50 | | 11 | 6.79 | |  |  |
|  | Asian | 445 | 17.18 | | 283 | 19.44 | | 38 | 16.89 | | 153 | 19.42 | | 29 | 17.90 | |  |  |
|  | Black | 238 | 9.19 | | 220 | 15.11 | | 30 | 13.33 | | 117 | 14.85 | | 23 | 14.20 | |  |  |
|  | Chinese | 70 | 2.70 | | 28 | 1.92 | | 3 | 1.33 | | 13 | 1.65 | | 4 | 2.47 | |  |  |
|  | Other | 91 | 3.51 | | 67 | 4.60 | | 12 | 5.33 | | 35 | 4.44 | | 13 | 8.02 | |  |  |
|  | Missing | 128 | 4.94 | | 67 | 4.60 | | 9 | 4.00 | | 33 | 4.19 | | 9 | 5.56 | |  |  |
| IMD decile | 1 | 203 | 7.84 | | 129 | 8.86 | | 18 | 8.00 | | 72 | 9.14 | | 13 | 8.02 | | 42.09 (40) | 0.38 |
|  | 2 | 612 | 23.63 | | 422 | 28.98 | | 58 | 25.78 | | 221 | 28.05 | | 49 | 30.25 | |  |  |
|  | 3 | 539 | 20.81 | | 290 | 19.92 | | 50 | 22.22 | | 150 | 19.04 | | 36 | 22.22 | |  |  |
|  | 4 | 342 | 13.20 | | 171 | 11.74 | | 26 | 11.56 | | 94 | 11.93 | | 12 | 7.41 | |  |  |
|  | 5 | 306 | 11.81 | | 132 | 9.07 | | 24 | 10.67 | | 88 | 11.17 | | 21 | 12.96 | |  |  |
|  | 6 | 214 | 8.26 | | 108 | 7.42 | | 21 | 9.33 | | 60 | 7.61 | | 9 | 5.56 | |  |  |
|  | 7 | 140 | 5.41 | | 83 | 5.70 | | 12 | 5.33 | | 44 | 5.58 | | 9 | 5.56 | |  |  |
|  | 8 | 116 | 4.48 | | 58 | 3.98 | | 6 | 2.67 | | 27 | 3.43 | | 3 | 1.85 | |  |  |
|  | 9 | 53 | 2.05 | | 28 | 1.92 | | 4 | 1.78 | | 15 | 1.90 | | 6 | 3.70 | |  |  |
|  | 10 | 27 | 1.04 | | 13 | 0.89 | | 1 | 0.44 | | 5 | 0.63 | | 1 | 0.62 | |  |  |
|  | Missing | 38 | 1.47 | | 22 | 1.51 | | 5 | 2.22 | | 12 | 1.52 | | 3 | 1.85 | |  |  |
| Sexual orientation | Heterosexual | 1,761 | 67.99 | | 1,017 | 69.85 | | 164 | 72.89 | | 548 | 69.54 | | 117 | 72.22 | | 8.52 (12) | 0.743 |
|  | Gay/Lesbian | 89 | 3.44 | | 45 | 3.09 | | 10 | 4.44 | | 25 | 3.17 | | 8 | 4.94 | |  |  |
|  | Bi-sexual | 153 | 5.91 | | 82 | 5.63 | | 12 | 5.33 | | 46 | 5.84 | | 9 | 5.56 | |  |  |
|  | Missing | 587 | 22.66 | | 312 | 21.43 | | 39 | 17.33 | | 169 | 21.45 | | 28 | 17.28 | |  |  |
| Medication | Prescribed-not taking | 114 | 4.40 | | 84 | 5.77 | | 8 | 3.56 | | 30 | 3.81 | | 6 | 3.70 | | 25.80 (12) | 0.011 |
|  | Prescribed and taking | 596 | 23.01 | | 379 | 26.03 | | 63 | 28.00 | | 200 | 25.38 | | 39 | 24.07 | |  |  |
|  | Not prescribed | 1,742 | 67.26 | | 894 | 61.40 | | 148 | 65.78 | | 520 | 65.99 | | 105 | 64.81 | |  |  |
|  | Missing | 138 | 5.33 | | 99 | 6.80 | | 6 | 2.67 | | 38 | 4.82 | | 12 | 7.41 | |  |  |
| Long term condition | No | 1,776 | 68.57 | | 935 | 64.22 | | 155 | 68.89 | | 550 | 69.80 | | 110 | 67.90 | | 13.34 (8) | 0.101 |
|  | Yes | 377 | 14.56 | | 236 | 16.21 | | 35 | 15.56 | | 106 | 13.45 | | 29 | 17.90 | |  |  |
|  | Missing | 437 | 16.87 | | 285 | 19.57 | | 35 | 15.56 | | 132 | 16.75 | | 23 | 14.20 | |  |  |
| Problem Descriptor | Depression | 935 | 36.10 | | 629 | 43.20 | | 84 | 37.33 | | 345 | 43.78 | | 79 | 48.77 | | 156.54 (32) | <0.001 |
|  | Mixed A.D. | 143 | 5.52 | | 85 | 5.84 | | 20 | 8.89 | | 31 | 3.93 | | 8 | 4.94 | |  |  |
|  | GAD | 486 | 18.76 | | 164 | 11.26 | | 38 | 16.89 | | 124 | 15.74 | | 31 | 19.14 | |  |  |
|  | OCD | 125 | 4.83 | | 43 | 2.95 | | 11 | 4.89 | | 17 | 2.16 | | 4 | 2.47 | |  |  |
|  | PTSD | 58 | 2.24 | | 42 | 2.88 | | 5 | 2.22 | | 20 | 2.54 | | 4 | 2.47 | |  |  |
|  | Other phobia and panic | 155 | 5.98 | | 83 | 5.70 | | 13 | 5.78 | | 51 | 6.47 | | 6 | 3.70 | |  |  |
|  | Social phobia | 119 | 4.59 | | 155 | 10.65 | | 23 | 10.22 | | 49 | 6.22 | | 6 | 3.70 | |  |  |
|  | Unspecified anxiety | 123 | 4.75 | | 60 | 4.12 | | 7 | 3.11 | | 35 | 4.44 | | 6 | 3.70 | |  |  |
|  | Missing | 446 | 17.22 | | 195 | 13.39 | | 24 | 10.67 | | 116 | 14.72 | | 18 | 11.11 | |  |  |
| Treatment outcomes | Reliable recovery | 1,348 | 52.05 | | 364 | 25.00 | | 144 | 64.00 | | 499 | 63.32 | | 43 | 26.54 | | 445.96 (4) | <0.001 |
|  | Reliable improvement | 1,913 | 73.86 | | 871 | 59.82 | | 199 | 88.44 | | 670 | 85.03 | | 85 | 52.47 | | 236.23 (4) | <0.001 |
|  | Deterioration | 136 | 5.25 | | 143 | 9.82 | | 6 | 2.67 | | 24 | 3.05 | | 30 | 18.52 | | 92.58 (4) | <0.001 |
|  | Attrition | 682 | 28.12 | | 516 | 39.15 | | 25 | 11.57 | | 212 | 28.46 | | 52 | 37.41 | | 93.64 (4) | <0.001 |

## Appendix 7b) Descriptive statistics of classes of WSAS5 trajectories

| **Appendix 5.2b: Characteristics of WSAS-5 trajectory classes** | | | | | | | | | | | | |
| --- | --- | --- | --- | --- | --- | --- | --- | --- | --- | --- | --- | --- |
|  | | **WSAS-5 Close relationships** | | | | | | | | | | |
|  |  | **Class 1 (n=3,498)** | | | **Class 2 (n=1,565)** | | | **Class 3 (n=158)** | | | **F (df=4)** | **p** |
| **Continuous measures** | | **n** | **M** | **SD** | **n** | **M** | **SD** | **n** | **M** | **SD** |  |  |
| PHQ-9 | | 3,497 | 14.10 | 5.15 | 1,565 | 17.07 | 4.70 | 158 | 17.45 | 4.89 | 206.82 | <0.001 |
| GAD-7 | | 3,496 | 13.10 | 4.26 | 1,565 | 14.66 | 4.10 | 158 | 15.01 | 3.96 | 83.22 | <0.001 |
| WSAS-2 | | 3,446 | 3.00 | 2.22 | 1,545 | 4.11 | 2.36 | 157 | 4.02 | 2.33 | 134.96 | <0.001 |
| WSAS-3 | | 3,446 | 4.00 | 2.20 | 1,545 | 5.13 | 2.16 | 157 | 5.71 | 1.89 | 173.30 | <0.001 |
| WSAS-4 | | 3,445 | 3.21 | 2.33 | 1,545 | 4.17 | 2.44 | 157 | 4.58 | 2.41 | 102.33 | <0.001 |
| WSAS-5 | | 3,446 | 3.55 | 2.25 | 1,545 | 5.31 | 2.09 | 157 | 5.85 | 1.89 | 393.48 | <0.001 |
| Agoraphobia item | | 3,475 | 2.74 | 2.53 | 1,557 | 3.43 | 2.65 | 158 | 3.42 | 2.89 | 40.92 | <0.001 |
| Social phobia item | | 3,475 | 3.25 | 2.34 | 1,557 | 4.21 | 2.45 | 158 | 4.18 | 2.55 | 93.85 | <0.001 |
| Specific phobia item | | 3,475 | 2.21 | 2.51 | 1,556 | 2.71 | 2.68 | 158 | 2.67 | 2.85 | 20.87 | <0.001 |
| Number LI sessions | | 3,498 | 3.03 | 2.73 | 1,565 | 2.77 | 2.84 | 158 | 3.03 | 3.15 | 4.97 | 0.007 |
| Number HI sessions | | 3,498 | 4.73 | 5.16 | 1,565 | 6.36 | 5.84 | 158 | 6.01 | 4.77 | 51.19 | <0.001 |
| Weeks-referral to assessment | | 3,495 | 3.38 | 3.16 | 1,564 | 3.21 | 2.92 | 158 | 3.68 | 3.75 | 2.62 | 0.073 |
| Weeks-assessment to treatment | | 3,399 | 8.16 | 7.81 | 1,525 | 8.90 | 8.06 | 154 | 9.34 | 8.75 | 5.67 | 0.004 |
| Age | | 3,498 | 20.70 | 2.24 | 1,565 | 20.50 | 2.13 | 158 | 20.67 | 2.18 | 4.32 | 0.013 |
| **Categorical Measures** | | **N** | **%** | | **N** | **%** | | **N** | **%** | | **X2 (df)** | **p** |
| Gender | Male | 901 | 25.76 | | 400 | 25.56 | | 45 | 28.48 | | 4.00 (4) | 0.406 |
|  | Female | 2,588 | 73.99 | | 1,156 | 73.87 | | 112 | 70.89 | |  |  |
|  | Missing | 9 | 0.26 | | 9 | 0.58 | | 1 | 0.63 | |  |  |
| Ethnicity | White | 1,820 | 52.03 | | 725 | 46.33 | | 72 | 45.57 | | 33.94 (12) | 0.001 |
|  | Mixed | 307 | 8.78 | | 122 | 7.80 | | 17 | 10.76 | |  |  |
|  | Asian | 586 | 16.75 | | 329 | 21.02 | | 33 | 20.89 | |  |  |
|  | Black | 386 | 11.03 | | 221 | 14.12 | | 21 | 13.29 | |  |  |
|  | Chinese | 86 | 2.46 | | 30 | 1.92 | | 2 | 1.27 | |  |  |
|  | Other | 141 | 4.03 | | 71 | 4.54 | | 6 | 3.80 | |  |  |
|  | Missing | 172 | 4.92 | | 67 | 4.28 | | 7 | 4.43 | |  |  |
| IMD decile | 1 | 280 | 8.00 | | 139 | 8.88 | | 16 | 10.13 | | 18.87 (20) | 0.530 |
|  | 2 | 884 | 25.27 | | 436 | 27.86 | | 42 | 26.58 | |  |  |
|  | 3 | 715 | 20.44 | | 326 | 20.83 | | 24 | 15.19 | |  |  |
|  | 4 | 434 | 12.41 | | 186 | 11.88 | | 25 | 15.82 | |  |  |
|  | 5 | 404 | 11.55 | | 150 | 9.58 | | 17 | 10.76 | |  |  |
|  | 6 | 271 | 7.75 | | 130 | 8.31 | | 11 | 6.96 | |  |  |
|  | 7 | 200 | 5.72 | | 79 | 5.05 | | 9 | 5.70 | |  |  |
|  | 8 | 148 | 4.23 | | 57 | 3.64 | | 5 | 3.16 | |  |  |
|  | 9 | 73 | 2.09 | | 27 | 1.73 | | 6 | 3.80 | |  |  |
|  | 10 | 33 | 0.94 | | 13 | 0.83 | | 1 | 0.63 | |  |  |
|  | Missing | 56 | 1.60 | | 22 | 1.41 | | 2 | 1.27 | |  |  |
| Sexual orientation | Heterosexual | 2,399 | 68.58 | | 1,093 | 69.84 | | 115 | 72.78 | | 7.58 (6) | 0.271 |
|  | Gay/Lesbian | 113 | 3.23 | | 56 | 3.58 | | 8 | 5.06 | |  |  |
|  | Bi-sexual | 199 | 5.69 | | 92 | 5.88 | | 11 | 6.96 | |  |  |
|  | Missing | 787 | 22.50 | | 324 | 20.70 | | 24 | 15.19 | |  |  |
| Medication | Prescribed-not taking | 152 | 4.35 | | 84 | 5.37 | | 6 | 3.80 | | 5.05 (6) | 0.538 |
|  | Prescribed and taking | 838 | 23.96 | | 399 | 25.50 | | 40 | 25.32 | |  |  |
|  | Not prescribed | 2,312 | 66.09 | | 993 | 63.45 | | 104 | 65.82 | |  |  |
|  | Missing | 196 | 5.60 | | 89 | 5.69 | | 8 | 5.06 | |  |  |
| Long term condition | No | 2,388 | 68.27 | | 1,040 | 66.45 | | 98 | 62.03 | | 8.49 (4) | 0.075 |
|  | Yes | 513 | 14.67 | | 235 | 15.02 | | 35 | 22.15 | |  |  |
|  | Missing | 597 | 17.07 | | 290 | 18.53 | | 25 | 15.82 | |  |  |
| Problem Descriptor | Depression | 1,278 | 36.54 | | 727 | 46.45 | | 67 | 42.41 | | 97.17 (16) | <0.001 |
|  | Mixed A.D. | 175 | 5.00 | | 96 | 6.13 | | 16 | 10.13 | |  |  |
|  | GAD | 641 | 18.32 | | 182 | 11.63 | | 20 | 12.66 | |  |  |
|  | OCD | 143 | 4.09 | | 52 | 3.32 | | 5 | 3.16 | |  |  |
|  | PTSD | 78 | 2.23 | | 45 | 2.88 | | 6 | 3.80 | |  |  |
|  | Other phobia and panic | 242 | 6.92 | | 60 | 3.83 | | 6 | 3.80 | |  |  |
|  | Social phobia | 224 | 6.40 | | 115 | 7.35 | | 13 | 8.23 | |  |  |
|  | Unspecified anxiety | 164 | 4.69 | | 60 | 3.83 | | 7 | 4.43 | |  |  |
|  | Missing | 553 | 15.81 | | 228 | 14.57 | | 18 | 11.39 | |  |  |
| Treatment outcomes | Reliable recovery | 1,898 | 54.26 | | 399 | 25.50 | | 101 | 63.92 | | 381.48 (2) | <0.001 |
|  | Reliable improvement | 2,679 | 76.59 | | 919 | 58.72 | | 140 | 88.61 | | 192.87 (2) | <0.001 |
|  | Deterioration | 164 | 4.69 | | 173 | 11.05 | | 2 | 1.27 | | 79.50 (2) | <0.001 |
|  | Attrition | 919 | 27.79 | | 547 | 39.49 | | 21 | 13.91 | | 83.53 (2) | <0.001 |

| Appendix 8: Logistic regression models for the association between class assignment and treatment outcomes (models 1-4). | | | | | | | |
| --- | --- | --- | --- | --- | --- | --- | --- |
| **WSAS item** | **Model** |  | **Class (vs class 1)** | **Reliable Recovery** | **Reliable Improvement** | **Deterioration** | **Attrition** |
| WSAS-3: Social leisure activities | Model 1 | Trajectory class | Class 2 | 0.31 | 0.53 | 1.97 | 1.64 |
|  |  |  |  | (0.27 - 0.35) | (0.46 - 0.60) | (1.54 - 2.51) | (1.43 - 1.89) |
|  |  |  | Class 3 | 1.64 | 2.71 | 0.49 | 0.33 |
|  |  |  |  | (1.23 - 2.17) | (1.78 - 4.11) | (0.22 - 1.13) | (0.22 - 0.51) |
|  |  |  | Class 4 | 1.59 | 2.01 | 0.57 | 1.02 |
|  |  |  |  | (1.35 - 1.87) | (1.62 - 2.49) | (0.36 - 0.88) | (0.85 - 1.22) |
|  |  |  | Class 5 | 0.33 | 0.39 | 4.10 | 1.53 |
|  |  |  |  | (0.23 - 0.48) | (0.28 - 0.54) | (2.66 - 6.32) | (1.07 - 2.18) |
|  | Model 2 | + Service level variables* | Class 2 | 0.25 | 0.44 | 2.14 | 2.80 |
|  |  |  |  | (0.21 - 0.29) | (0.38 - 0.51) | (1.67 - 2.74) | (2.34 - 3.34) |
|  |  |  | Class 3 | 1.33 | 2.11 | 0.57 | 0.62 |
|  |  |  |  | (1.00 - 1.78) | (1.38 - 3.22) | (0.25 - 1.31) | (0.40 - 0.98) |
|  |  |  | Class 4 | 1.62 | 2.04 | 0.57 | 0.88 |
|  |  |  |  | (1.37 - 1.92) | (1.64 - 2.54) | (0.36 - 0.88) | (0.72 - 1.08) |
|  |  |  | Class 5 | 0.27 | 0.32 | 4.52 | 2.64 |
|  |  |  |  | (0.19 - 0.39) | (0.23 - 0.44) | (2.92 - 7.01) | (1.72 - 4.06) |
|  | Model 3 | + Baseline severity ǂ | Class 2 | 0.31 | 0.38 | 3.02 | 1.98 |
|  |  |  |  | (0.26 - 0.36) | (0.32 - 0.45) | (2.27 - 4.00) | (1.63 - 2.41) |
|  |  |  | Class 3 | 1.70 | 1.88 | 0.74 | 0.45 |
|  |  |  |  | (1.26 - 2.29) | (1.22 - 2.88) | (0.32 - 1.72) | (0.28 - 0.71) |
|  |  |  | Class 4 | 2.06 | 1.82 | 0.74 | 0.66 |
|  |  |  |  | (1.72 - 2.46) | (1.45 - 2.28) | (0.47 - 1.17) | (0.53 - 0.82) |
|  |  |  | Class 5 | 0.30 | 0.29 | 5.65 | 2.28 |
|  |  |  |  | (0.20 - 0.43) | (0.21 - 0.40) | (3.58 - 8.90) | (1.47 - 3.53) |
|  | Model 4 | + Demographic factors§ | Class 2 | 0.31 | 0.37 | 3.22 | 1.96 |
|  |  |  |  | (0.26 - 0.36) | (0.31 - 0.44) | (2.41 - 4.29) | (1.61 - 2.39) |
|  |  |  | Class 3 | 1.73 | 1.87 | 0.77 | 0.44 |
|  |  |  |  | (1.28 - 2.34) | (1.22 - 2.88) | (0.33 - 1.80) | (0.28 - 0.70) |
|  |  |  | Class 4 | 2.07 | 1.80 | 0.77 | 0.64 |
|  |  |  |  | (1.72 - 2.48) | (1.43 - 2.26) | (0.48 - 1.21) | (0.51 - 0.80) |
|  |  |  | Class 5 | 0.28 | 0.28 | 5.95 | 2.30 |
|  |  |  |  | (0.19 - 0.42) | (0.20 - 0.39) | (3.73 - 9.50) | (1.48 - 3.58) |
| WSAS-5: Close social relationships | Model 1 | Trajectory class | Class 2 | 0.29 | 0.43 | 2.53 | 1.70 |
|  |  |  |  | (0.25 - 0.33) | (0.38 - 0.49) | (2.02 - 3.16) | (1.49 - 1.94) |
|  |  |  | Class 3 | 1.49 | 2.38 | 0.26 | 0.42 |
|  |  |  |  | (1.07 - 2.08) | (1.45 - 3.91) | (0.06 - 1.06) | (0.26 - 0.67) |
|  | Model 2 | + Service level variables* | Class 2 | 0.24 | 0.37 | 2.74 | 2.82 |
|  |  |  |  | (0.21 - 0.28) | (0.32 - 0.42) | (2.18 - 3.44) | (2.39 - 3.33) |
|  |  |  | Class 3 | 1.32 | 2.00 | 0.28 | 0.69 |
|  |  |  |  | (0.94 - 1.84) | (1.21 - 3.31) | (0.07 - 1.15) | (0.42 - 1.13) |
|  | Model 3 | + Baseline severity ǂ | Class 2 | 0.28 | 0.32 | 3.79 | 2.29 |
|  |  |  |  | (0.25 - 0.33) | (0.27 - 0.36) | (2.96 - 4.85) | (1.93 - 2.72) |
|  |  |  | Class 3 | 1.67 | 1.70 | 0.40 | 0.52 |
|  |  |  |  | (1.19 - 2.36) | (1.02 - 2.81) | (0.10 - 1.64) | (0.31 - 0.86) |
|  | Model 4 | + Demographic factors § | Class 2 | 0.28 | 0.32 | 3.69 | 2.34 |
|  |  |  |  | (0.24 - 0.32) | (0.27 - 0.36) | (2.87 - 4.76) | (1.97 - 2.79) |
|  |  |  | Class 3 | 1.72 | 1.68 | 0.41 | 0.52 |
|  |  |  |  | (1.21 - 2.43) | (1.01 - 2.79) | (0.10 - 1.68) | (0.31 - 0.87) |
| * Number low intensity sessions, number high intensity sessions, weeks from referral to assessment, weeks from assessment to treatment, trust. | | | | | | | |
| ǂPHQ9, GAD7, phobias | | | | | | | |
| § IMD, age, gender ethnicity, diagnosis, long term conditions, medication use, sexual orientation. | | | | | | | |
| ⸹ N=5,221 for reliable recovery, reliable improvement and deterioration. N=4,843 for attrition | | | | | | | |

| Appendix 9: Logistic regression analysis using complete cases only | | | | | | | | | | |
| --- | --- | --- | --- | --- | --- | --- | --- | --- | --- | --- |
| **WSAS item** | **Model** |  | | **Class (vs class 1)** | **Reliable Recovery** | **Reliable Improvement** | **Deterioration** | | **Attrition** | |
| WSAS-3: Social Leisure activities | Model 1 *(n= 5,221 for reliable recovery, reliable improvement and deterioration. n= 4,843 for attrition.*) | | Trajectory class | Class 2 | 0.31 | 0.53 | 1.97 | 1.64 | |  |
|  |  |  |  |  | (0.27 - 0.35) | (0.46 - 0.60) | (1.54 - 2.51) | (1.43 - 1.89) | |  |
|  |  |  |  | Class 3 | 1.64 | 2.71 | 0.49 | 0.33 | |  |
|  |  |  |  |  | (1.23 - 2.17) | (1.78 - 4.11) | (0.22 - 1.13) | (0.22 - 0.51) | |  |
|  |  |  |  | Class 4 | 1.59 | 2.01 | 0.57 | 1.02 | |  |
|  |  |  |  |  | (1.35 - 1.87) | (1.62 - 2.49) | (0.36 - 0.88) | (0.85 - 1.22) | |  |
|  |  |  |  | Class 5 | 0.33 | 0.39 | 4.10 | 1.53 | |  |
|  |  |  |  |  | (0.23 - 0.48) | (0.28 - 0.54) | (2.66 - 6.32) | (1.07 - 2.18) | |  |
|  | Model 2 *(n= 5,074 for reliable recovery, reliable improvement and deterioration. n= 4,711 for attrition.*) | | + Service level variables* | Class 2 | 0.25 | 0.44 | 2.08 | 2.79 | |  |
|  |  |  |  |  | (0.22 - 0.29) | (0.38 - 0.51) | (1.61 - 2.68) | (2.34 - 3.34) | |  |
|  |  |  |  | Class 3 | 1.30 | 2.07 | 0.57 | 0.62 | |  |
|  |  |  |  |  | (0.97 - 1.74) | (1.36 - 3.17) | (0.25 - 1.32) | (0.39 - 0.98) | |  |
|  |  |  |  | Class 4 | 1.62 | 2.06 | 0.54 | 0.89 | |  |
|  |  |  |  |  | (1.37 - 1.93) | (1.65 - 2.57) | (0.34 - 0.85) | (0.72 - 1.10) | |  |
|  |  |  |  | Class 5 | 0.26 | 0.32 | 4.49 | 2.68 | |  |
|  |  |  |  |  | (0.18 - 0.38) | (0.23 - 0.45) | (2.87 - 7.00) | (1.73 - 4.14) | |  |
|  | Model 3 *(n=5,042 for reliable recovery, reliable improvement and deterioration. n= 4,680 for attrition.)* | | + Baseline severity ǂ | Class 2 | 0.31 | 0.38 | 2.91 | 1.97 | |  |
|  |  |  |  |  | (0.26 - 0.37) | (0.32 - 0.45) | (2.18 - 3.88) | (1.62 - 2.41) | |  |
|  |  |  |  | Class 3 | 1.66 | 1.86 | 0.73 | 0.45 | |  |
|  |  |  |  |  | (1.23 - 2.25) | (1.21 - 2.86) | (0.31 - 1.70) | (0.28 - 0.71) | |  |
|  |  |  |  | Class 4 | 2.05 | 1.84 | 0.70 | 0.66 | |  |
|  |  |  |  |  | (1.70 - 2.45) | (1.46 - 2.32) | (0.43 - 1.12) | (0.53 - 0.82) | |  |
|  |  |  |  | Class 5 | 0.29 | 0.29 | 5.52 | 2.29 | |  |
|  |  |  |  |  | (0.20 - 0.43) | (0.21 - 0.41) | (3.48 - 8.77) | (1.47 - 3.57) | |  |
|  | Model 4 *(n=5,042 for reliable recovery, reliable improvement and deterioration. n= 4,680 for attrition.)* | | + Demographic factors§ | Class 2 | 0.31 | 0.37 | 3.11 | 1.96 | |  |
|  |  |  |  |  | (0.26 - 0.37) | (0.32 - 0.44) | (2.32 - 4.17) | (1.60 - 2.39) | |  |
|  |  |  |  | Class 3 | 1.69 | 1.86 | 0.76 | 0.44 | |  |
|  |  |  |  |  | (1.24 - 2.29) | (1.20 - 2.86) | (0.33 - 1.78) | (0.27 - 0.71) | |  |
|  |  |  |  | Class 4 | 2.05 | 1.82 | 0.72 | 0.64 | |  |
|  |  |  |  |  | (1.70 - 2.46) | (1.44 - 2.29) | (0.44 - 1.15) | (0.51 - 0.80) | |  |
|  |  |  |  | Class 5 | 0.28 | 0.29 | 5.86 | 2.31 | |  |
|  |  |  |  |  | (0.19 - 0.41) | (0.20 - 0.40) | (3.64 - 9.43) | (1.48 - 3.61) | |  |
| WSAS-5: Close relationships | Model 1 *(n= 5,221 for reliable recovery, reliable improvement and deterioration. n= 4,843 for attrition.*) | | Trajectory class | Class 2 | 0.29 | 0.43 | 2.53 | 1.70 | |  |
|  |  |  |  |  | (0.25 - 0.33) | (0.38 - 0.49) | (2.02 - 3.16) | (1.49 - 1.94) | |  |
|  |  |  |  | Class 3 | 1.49 | 2.38 | 0.26 | 0.42 | |  |
|  |  |  |  |  | (1.07 - 2.08) | (1.45 - 3.91) | (0.06 - 1.06) | (0.26 - 0.67) | |  |
|  | Model 2 *(n= 5,074 for reliable recovery, reliable improvement and deterioration. n= 4,711 for attrition.*) | | + Service level variables* | Class 2 | 0.24 | 0.37 | 2.76 | 2.78 | |  |
|  |  |  |  |  | (0.21 - 0.28) | (0.32 - 0.42) | (2.19 - 3.48) | (2.36 - 3.29) | |  |
|  |  |  |  | Class 3 | 1.34 | 2.08 | 0.29 | 0.65 | |  |
|  |  |  |  |  | (0.96 - 1.89) | (1.24 - 3.48) | (0.07 - 1.19) | (0.39 - 1.09) | |  |
|  | Model 3 *(n=5,042 for reliable recovery, reliable improvement and deterioration. n= 4,680 for attrition.)* | | + Baseline severity ǂ | Class 2 | 0.29 | 0.31 | 3.86 | 2.28 | |  |
|  |  |  |  |  | (0.25 - 0.33) | (0.27 - 0.36) | (3.00 - 4.97) | (1.92 - 2.71) | |  |
|  |  |  |  | Class 3 | 1.71 | 1.74 | 0.42 | 0.48 | |  |
|  |  |  |  |  | (1.21 - 2.42) | (1.03 - 2.92) | (0.10 - 1.74) | (0.29 - 0.82) | |  |
|  | Model 4 *(n=5,042 for reliable recovery, reliable improvement and deterioration. n= 4,680 for attrition.)* | | + Demographic factors § | Class 2 | 0.28 | 0.31 | 3.75 | 2.32 | |  |
|  |  |  |  |  | (0.24 - 0.33) | (0.27 - 0.36) | (2.90 - 4.86) | (1.95 - 2.77) | |  |
|  |  |  |  | Class 3 | 1.76 | 1.72 | 0.43 | 0.49 | |  |
|  |  |  |  |  | (1.24 - 2.51) | (1.02 - 2.90) | (0.10 - 1.77) | (0.29 - 0.83) | |  |
| * Number low intensity sessions, number high intensity sessions, weeks from referral to assessment, weeks from assessment to treatment, trust. | | | | | | | | | |  |
| ǂPHQ9, GAD7, phobias | | | | | | | | | |  |
| § IMD, age, gender ethnicity, diagnosis, long term conditions, medication use, sexual orientation. | | | | | | | | | |  |

# Appendix 10: comparisons between classes who improved and classes who remained impaired in associations with treatment outcomes: fully adjusted models with imputed data

|  | | | | | | |
| --- | --- | --- | --- | --- | --- | --- |
| **WSAS item** | **Model** | **Class (vs class 2)** | **Reliable Recovery** | **Reliable Improvement** | **Deterioration** | **Attrition** |
| WSAS-3: Social Leisure activities | Model 4* | Class 3 | 5.59 | 4.99 | 0.25 | 0.22 |
|  |  |  | (4.09 - 7.64) | (3.24 - 7.69) | (0.11 - 0.57) | (0.14 - 0.36) |
|  |  | Class 4 | 6.62 | 4.8 | 0.24 | 0.32 |
|  |  |  | (5.40 - 8.13) | (3.80 - 6.07) | (0.15 - 0.39) | (0.25 - 0.41) |
| WSAS-5: Close relationships | Model 4* | Class 3 | 6.05 | 5.19 | 0.11 | 0.22 |
|  |  |  | (4.22 - 8.67) | (3.12 - 8.64) | (0.03 - 0.46) | (0.13 - 0.38) |
| Note: N=5,221 for reliable recovery, reliable improvement and deterioration. N=4,843 for attrition  * Adjusted for number low intensity sessions, number high intensity sessions, weeks from referral to assessment, weeks from assessment to treatment, trust, PHQ9, GAD7, phobias, IMD, age, gender ethnicity, diagnosis, long term conditions, medication use, sexual orientation. | | | | | | |

# Appendix 11: comparisons between classes who improved and classes who remained impaired in associations with treatment outcomes: fully adjusted models with complete cases only

| **WSAS item** | **Model** | **Class (vs class 2)** | **Reliable Recovery** | **Reliable Improvement** | **Deterioration** | **Attrition** |
| --- | --- | --- | --- | --- | --- | --- |
| WSAS-3: Social Leisure activities | Model 4* | Class 3 | 5.32 | 4.85 | 0.25 | 0.23 |
|  |  |  | (3.88 - 7.31) | (3.14 - 7.48) | (0.11 - 0.59) | (0.14 - 0.37) |
|  |  | Class 4 | 6.53 | 4.82 | 0.23 | 0.32 |
|  |  |  | (5.30 - 8.05) | (3.79 - 6.13) | (0.14 - 0.37) | (0.25 - 0.41) |
| WSAS-5: Close relationships | Model 4* | Class 3 | 6.13 | 5.34 | 0.12 | 0.21 |
|  |  |  | (4.25 - 8.83) | (3.16 - 9.01) | (0.03 - 0.48) | (0.12 - 0.36) |
| Note: N=4,992 for reliable recovery, reliable improvement and deterioration. N=4,634 for attrition  * Adjusted for number low intensity sessions, number high intensity sessions, weeks from referral to assessment, weeks from assessment to treatment, trust, PHQ9, GAD7, phobias, IMD, age, gender ethnicity, diagnosis, long term conditions, medication use, sexual orientation. | | | | | | |

# Appendices References

Bentler, P. M. (1990). Comparative fit indexes in structural models. *Psychological bulletin, 107*(2), 238.

Bentler, P. M., & Bonett, D. G. (1980). Significance tests and goodness of fit in the analysis of covariance structures. *Psychological bulletin, 88*(3), 588.

Bollen, K. A., & Curran, P. J. (2006). *Latent curve models: A structural equation perspective*: John Wiley & Sons.

Browne, M. W., & Cudeck, R. (1992). Alternative ways of assessing model fit. *Sociological methods & research, 21*(2), 230-258.

Clark, D. M. (2018). Realizing the mass public benefit of evidence-based psychological therapies: the IAPT program. *Annual review of clinical psychology, 14*, 159-183.

Clark, D. M., Canvin, L., Green, J., Layard, R., Pilling, S., & Janecka, M. (2018). Transparency about the outcomes of mental health services (IAPT approach): an analysis of public data. *The Lancet, 391*(10121), 679-686.

Dempster, A. P., Laird, N. M., & Rubin, D. B. (1977). Maximum likelihood from incomplete data via the EM algorithm. *Journal of the Royal Statistical Society: Series B (Methodological), 39*(1), 1-22.

Hu, L. t., & Bentler, P. M. (1999). Cutoff criteria for fit indexes in covariance structure analysis: Conventional criteria versus new alternatives. *Structural equation modeling: a multidisciplinary journal, 6*(1), 1-55.

Lo, Y., Mendell, N. R., & Rubin, D. B. (2001). Testing the number of components in a normal mixture. *Biometrika, 88*(3), 767-778.

Marks, I. M. (1986). *Behavioural psychotherapy: Maudsley pocket book of clinical management*: Wright/IOP Publishing.

McLachlan, G., & Peel, D. (2000). Finite mixture models. In. New York: Wiley.

Mundt, J. C., Marks, I. M., Shear, M. K., & Greist, J. M. (2002). The Work and Social Adjustment Scale: a simple measure of impairment in functioning. *The British Journal of Psychiatry, 180*(5), 461-464.

Muthén, L., & Muthén, B. (1998-2017). MPlus User's Guide.

Nylund, K. L., Asparouhov, T., & Muthén, B. O. (2007). Deciding on the number of classes in latent class analysis and growth mixture modeling: A Monte Carlo simulation study. *Structural equation modeling: a multidisciplinary journal, 14*(4), 535-569.

Steiger, J. H., & Lind, J. M. (1980). *Statistically based tests for the number of common factors.* Paper presented at the the annual meeting of the Psychometric Society. Iowa City, IA. 1980.

Tucker, L. R., & Lewis, C. (1973). A reliability coefficient for maximum likelihood factor analysis. *Psychometrika, 38*(1), 1-10.

Wickrama, K. A., Lee, T. K., O'Neal, C. W., & Lorenz, F. O. (2021). *Higher-order growth curves and mixture modeling with Mplus: A practical guide*: Routledge.
